# Rapid detection of *Escherichia coli* using bacteriophage-induced lysis and image analysis

**DOI:** 10.1371/journal.pone.0233853

**Published:** 2020-06-05

**Authors:** Xu Yang, Nicharee Wisuthiphaet, Glenn M. Young, Nitin Nitin

**Affiliations:** 1 Department of Food Science and Technology, University of California, Davis, Davis, California, United States of America; 2 Department of Biological and Agricultural Engineering, University of California, Davis, Davis, California, United States of America; The Pennsylvania State University, UNITED STATES

## Abstract

Rapid detection of bacterial pathogens is a critical unmet need for both food and environmental samples such as irrigation water. As a part of the Food safety Modernization Act (FSMA), The Produce Safety rule has established several requirements for testing for the presence of generic *Escherichia coli* in water, but the current method available for testing (EPA M1603) demands specified multiple colony verification and highly trained personnel to perform these tests. The purpose of the study was to assess a phage induced bacterial lysis using quantitative image analysis to achieve rapid detection of *E*. *coli* at low concentrations within 8 hours. This study aimed to develop a simple yet highly sensitive and specific approach to detect target bacteria in complex matrices. In the study, *E*. *coli* cells were first enriched in tryptic soy broth (TSB), followed by T7 phage induced lysis, concentration, staining and fluorescent imaging. Image analysis was conducted including image pre-processing, image segmentation and quantitatively analysis of cellular morphological features (area, eccentricity and full width at half maximum). Challenge experiments using realistic matrices, including simulated fresh produce wash water, coconut water and spinach wash water, demonstrated the method can be applied for use in situations that occur in food processing facilities. The results indicated *E*. *coli* cells that are lysed by T7 phages demonstrated significantly (P < 0.05) higher extracellular DNA release, altered cellular shape (from rod to circular) and diffused fluorescent signal intensity. Using this biosensing strategy, a sensitivity to detect *Escherichia coli* at 10 CFU/ml within 8 hours was achieved, both in laboratory medium and in complex matrices. The proposed phage based biosensing strategy enables rapid detection of bacteria and is applicable to analysis of food systems. Furthermore, the steps involved in this assay can be automated to enable detection of target bacteria in food facilities without extensive resources.

## Introduction

As part of the Food Safety Modernization Act (FSMA), The Produce Safety rule has established several standards for testing for the presence of generic *Escherichia coli* in water. For instance, no *E*. *coli* shall be detected in water that is directly used to contact any fresh produce after harvest or food-contact surfaces. In addition, agricultural water that is applied for irrigation produce crops should contain equal or less than 126 colony forming unit (CFU) per 100 ml of tested water [1]. To comply with The Produce Safety rule, sensitive, cost-effective and rapid methods for *E*. *coli* detection are desired. Ideally, a method for *E*. *coli* detection should be completed within 8-hours to match the typical work-shift schedule of most fresh produce packaging operations with a product that has a relatively short shelf-life [2]. The current method for generic *E*. *coli* detection in agricultural water is based on the U.S. Environmental Protection Agency Method 1603 (EPA M1603). This method requires specified multiple colony verification and highly trained personnel to perform a test. Furthermore, there is significant subjectivity in evaluating the false positive results [3].

Complementary to conventional detection approaches, lytic bacteriophages (phages) have also been evaluated as a bio-sensing element for the detection of bacteria. The extraordinary host specificity of phages for their host provides a naturally occurring event that can be co-opted into a method for bacterial pathogen detection [4]. In addition, the rapidity of phage multiplication provides a "built-in" amplification step that can be detected within a time-frame of hours [5]. Based on these advantages, a variety of phage-based biosensing technologies have been developed. Phage typing is a classical phage-based culture method for detecting specific bacterial pathogens. The formation of a visually observed clear plaque indicates the presence of a bacterial host specific to the phages supplemented [6]. The optical approach of detection involves monitoring the decrease of turbidity by using a spectrophotometer [7]. However, both visual and optical methods need relatively high number of host cells, which could be time consuming. Detection of phage-bacteria complex formation is another phage-based biosensing strategy. The phage-bacteria complex forms upon infection with high specificity and stability. One approach to detect the complex is to fluorescently label phages, followed by absorption phages onto bacteria. Then, by using flow cytometry or fluorescent microscopy, phage-bacteria complex can be detected [8–10]. The assay is simple and straightforward, but the fluorescent signal from only phages has a low signal-to-noise ratio [11]. Another phage-based biosensing technique is through utilization of reporter phages, which carry genetically modified reporter genes to manipulate host bacteria metabolic process [5]. Several studies have exploited this concept to develop genetically modified reporter phages to overexpress *β*-galactosidase or alkaline phosphatase [12–14]. However, the current approaches based on reporter phages may add more complexity and capital cost to the detection process.

As described above, a variety of phage-based biosensing approaches have been developed, but each method has some limitations. The current study was aimed at developing a novel, rapid and sensitive detection for *E*. *coli* through microscopy and image analysis. Imaging using fluorescence microscopy combined with automated image analysis is a promising alternative approach that allows improved sensitivity and reduced complexity of bacteria detection method protocols.

Microscopic imaging is able to focus on the fluorescence signal from an individual bacterial cell; therefore, it provides a single-cell level of detection sensitivity [15]. Implementing imaging and image analysis for bacteria detection has a potential to develop detection methods that require simple instrumental setup such as fluorescence microscope or even the miniaturized versions of microscope for field detection. The imaging procedure and image analysis can be automated which allow simple and user-friendly detection protocol with no specialized trained personnel required, and the simplified protocol results in more time-effective detection.

Moreover, imaging and image analysis process can be performed in the field since the aseptic technique environment is not required, unlike nucleic acid-based detection approaches which need to be conducted in a molecular laboratory. In the food industry, imaging and image analysis may not be commonly employed in food safety and quality control assay, but this approach has been widely used for medical diagnostic purposes. Currently, imaging procedures have been applied to detect that malaria parasite, *Plasmodium*, infection of red blood cells, based on cellular morphology using a simple cellphone-based microscopy which applicable to low-cost in-the-field optical diagnostics of malaria [16].

**Fig 1:**
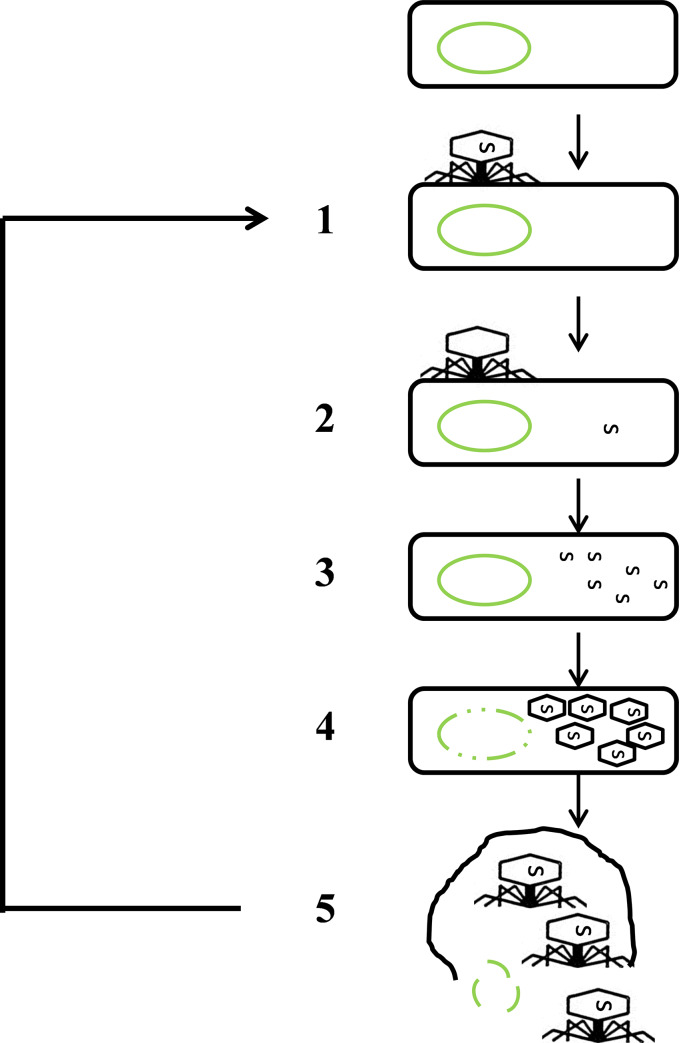
Life cycle of lytic T7 phages. Step 1, attachment of T7 phage on host; step 2, phage DNA injection into bacterial host; step 3, phage hijack bacteria metabolism and multiplication of phage DNA; step 4, production of new capsids and assembly of phages; step 5, host cell lysis and eDNA release.

The biosensing strategy in this study focuses on detection of *E*. *coli* through phage-induced lysis in authentic food samples and artificial wash water supplemented with organic chemicals simulating a chemical oxygen demand (COD) of wash water. Phages are well characterized for producing endolysin which could induce explosive host cell lysis [17]. As shown in Fig 1, the life cycle of lytic T7 phages starts when the specialized adsorption structures, called T7 tail fibers, bind to receptor molecules on *E*. *coli* BL21 (Step 1). Injection of phage DNA occurs when the phage tail tube punctures the membrane layers of *E*. *coli*, which is followed by injection of the viral genome into the cytoplasm of the host cell (step 2) [18,19]. Immediately after injection of the T7 phage genome, replication of the phage genome occurs; a process that involved co-opting of host cellular machinery. During this stage numerous copies of the phage genomic DNA are synthesized (step 3). Subsequently, protein subunits of the phage particle are synthesized and assemble into procapsids. During the final step a copy of the phage genomic DNA is packaged procapsids (step 4). Mature phages are then liberated by host cell lysis with the aid of two specific enzymes, holin and lysin. Bacterial host cells that undergo phage-induced lysis in Fig 1 illustrate a significant change in their cellular morphology, proposed as “rod-to-round” transition [20], followed by intracellular to extracellular DNA release, defined as environmental DNA (eDNA). Cellular morphology changes and eDNA release from host bacteria can be visualized under a microscope, coupled with a simple DNA stains and further fluorescence image analysis using BacFormatics v0.7 developed in MATLAB [20]. The key advantage of fluorescence image analysis is the higher signal-to-noise ratio and increased detection sensitivity, which hypothetically allows low detection limit at low concentrations of *E*. *coli* in a variety of different food matrices.

## Materials and methods

### Chemical reagents

SYBR Green I nucleic acid stain, 10,000× concentrate was purchased from Invitrogen, USA, and a working solution of 10× SYBR Green I was prepared in Milli-Q water. *p*-Phenylenediamine and chloroform were obtained from Acros Organic (Fair Lawn, NJ, USA) and a 10% (wt/vol) stock solution of *p*-phenylenediamine was prepared in Milli-Q water. Whatman® anodisc inorganic filter membrane (13 mm, 0.02 μm pore size) was obtained from GE Healthcare (Buckinghamshire, UK). Microscopic slide was obtained from VWR international (Radnor, PA, USA). Tryptic soy broth (TSB) and tryptic soy agar (TSA) were obtained from Sigma-Aldrich (St. Louis, MO, USA). Filtration system, cover glass and Luria Bertani (LB) broth were obtained from Fisher Scientific (Pittsburgh, PA, USA). Phosphate buffered saline (PBS) was purchased from Fisher Bioreagents (Fair Lawn, NJ, USA). Polycarbonate filter (20 um pore size, 47 mm diameter) was purchased from Maine Manufacturing (ME, USA). Milli-Q water was produced by QPAK^®^ 2 purification system (EMD Millipore, Billerica, MA, USA).

### Bacterial cultures and phage preparation

Both *E*. *coli* BL21 (ATCC BAA-1025) and bacteriophage T7 (ATCC BAA-1025-B2) were obtained from American type culture collection. *E*. *coli* BL21 was cultured in TSB broth at 37°C for 16 hours before use.

Bacteriophages were propagated as the following procedure. Bacteriophages were first inoculated into log-phase *E*. *coli* BL21 culture at the ratio of 1:100 (phage:bacteria). The mixture was then incubated at 37°C for 15 min for initial infection and then centrifuged at 16100 × *g* for 10 min. Supernatant was discarded and the same volume of TSB was added to resuspend the pellet, followed by incubating at 37°C with 200 rpm shaking until no visible turbidity was observed. Chloroform was then added to the final concentration at 20% (vol/vol) and incubated at 4°C overnight. Then, chloroform added mixture was centrifuged at 5,000 × *g* for 10 min and water phase was collected. The water phase which contained free phages, TSB medium and bacterial host lysis debris was then centrifuged again at 16100 × *g* for 10 min. Pellet was washed for one time and resuspended in PBS at the population of 10^9^ PFU/ml phage concentration.

### T7 phage induced *E*. *coli* cell lysis

T7 phage induced *E*. *coli* cell lysis was conducted in two modes: low titer co-incubation lysis (LTCL) and high titer two-step lysis (HTTL). LTCL refers to co-incubation of low initial phage concentration (10^2^ PFU/ml) with *E*. *coli* in TSB. LTCL allows for initial growth of *E*. *coli* with delayed lysis. HTTL refers to enriching *E*. *coli* first in TSB, followed by inoculation of high titer phage concentration (10^7^ PFU/ml) to induce lysis for 20 min.

### *E*. *coli* cellular morphological change and eDNA release during LTCL

The dynamic morphological change of *E*. *coli* and release of eDNA were analyzed through LTCL during extended lysis period as reported earlier [7,21]. Briefly, overnight *E*. *coli* BL21 cultures were precipitated, washed and resuspended in PBS. A 50-ml falcon tube containing 10 ml TSB pre-warmed at 37°C was prepared and inoculated with 10^3^ CFU/ml *E*. *coli* BL21 and 10^2^ PFU/ml T7 phage. The inoculated tube was then incubated at 37°C in shaking incubator for 2, 3 and 4 hours and 2 ml of the aliquot was taken at each time point, respectively. Then, aliquots from each time point were filtered through anodiscs and subsequently stained with SYBR Green I. The filtration and staining process were conducted according to published protocol [22]. Briefly, the filtration system was made up of a filtering flask, a fritted glass base and a polycarbonate filter. The anodisc filter was directly located on top of the polycarbonate filter, with the glass base and filtering flask attached underneath. Aliquot from each tube was gently pipetted onto their respective anodisc for filtration. After filtration, anodisc was carefully removed from the polycarbonate filter onto a microscopic slide pre-spotted with 20 μl of SYBR Green I stock solution. The anodisc was then transferred directly onto the SYBR Green I drop with backside down since the stain can easily pass through the anodisc to stain the microorganisms on the topside. The anodisc was later stained in a dark laboratory bench drawer for 20 min before the dye was removed by gently rubbing the backside anodisc filter against a Kimwipe. In the meanwhile, 1% of *p*-phenylenediamine was prepared from the 10% stock solution as anti-fading reagent. After staining and removal of excess dyes, the anodisc was transferred directly onto a new microscopic slide with 20 μl anti-fading reagent drop. The anodisc was eventually covered with a cover glass and observed under an Olympus IX-71 inverted research fluorescence microscope with a ×100 (1.25 NA) objective lens, a CCD (charge coupled device) camera (Model C4742-80-12AG, Hamamatsu, Tokyo, Japan) and Metamorph imaging software (version 7.7.2.0, Universal Imaging Corporation). An average of 10–12 images were taken for each anodisc sample. The fluorescence excitation/emission wavelength of SYBR Green I stain was 480 ± 30 and 535 ± 40 nm, respectively. Negative controls were performed at the same condition except for adding phages. All conditions including negative controls were performed in triplicates.

### Characterization of *E*. *coli* morphological change and eDNA release through LTCL

Similar experimental procedure was followed as described in the previous section. Three 50 ml falcon tubes (A, B, and C) containing 10 ml TSB were prepared and inoculated with 10^2^ PFU/ml T7 phages in each tube. Tube A received *E*. *coli* inoculation at 10^2^ CFU/ml, tube B received *E*. *coli* inoculation at 10^3^ CFU/ml while tube C received *E*. *coli* inoculation at 10^4^ CFU/ml. All tubes were then incubated at 37°C with 200 rpm shaking for 2, 3 and 4 hours, respectively. After incubation, contents from each tube were filtered onto their respective anodisc, and subsequently stained and observed under fluorescence microscope as described earlier. Negative controls were conducted at the same condition without phage inoculation. Both phage supplemented groups and negative controls were performed in triplicates.

### Characterization of *E*. *coli* morphological change and eDNA release through HTTL

To start with, three 50 ml falcon tubes (A, B, and C) each containing 5 ml of TSB were prepared and pre-warmed in 37°C water bath before inoculation. Overnight *E*. *coli* BL21 cultures were precipitated, washed and resuspended in PBS, followed by inoculation in tube A, B and C at 10^1^, 10^2^ and 10^3^ CFU/ml, respectively. All tubes were then incubated at 37°C with 200 rpm shaking for 5, 4 and 2 hours, respectively. After incubation, T7 phages were inoculated in all tubes at 10^7^ PFU/ml, followed by lysis at the same condition for 20 min. Then, aliquots from each tube were filtered through anodiscs, and were subsequently stained with SYBR Green and observed under the microscope as described earlier. Negative controls were performed at the same condition without phage addition. All conditions including negative controls were performed in triplicates.

### Validation of the biosensing approach in realistic food items

HTTL was chosen to be a more robust, consistent and sensitive approach for analyzing morphological change and eDNA release of *E*. *coli* in realistic food matrices, including artificial wash water, coconut water and spinach wash water. Coconut water was selected as a challenging matrix as it contains sugars, amino acids, vitamins and phytohormones [23]. Artificial wash water was created by adding LB broth to sterile water to achieve a final COD at 1,000 ppm, which is comparable to fresh-cut produce wash water in industry [24]. Both coconut water and artificial wash water were inoculated with *E*. *coli* at 10 CFU/ml, followed by mixing with double strength TSB at 1:1 (vol/vol) ratio for pre-enrichment. The mixture was then shaking incubated at 37°C for 5 hours and subsequently lysed with 10^7^ PFU/ml T7 phages for 20 min. Content after lysis was filtered onto anodisc, stained and visualized under a microscope. Negative control without phage lysis was also conducted and both conditions were performed in triplicates. For spinach wash water, detailed sample preparation is described in the supporting information.

### Image analysis—preprocessing

All images were subjected to analysis by Matlab^TM^ 2017a software (Mathworks, Natick, Mass., USA) to generate binary images, followed by quantification of morphological features. Before binary images conversion, a series of image pre-processing functions were applied to enhance image visual experience, remove uneven background illumination and adjust for fluctuation in data acquisition. Specifically, to best represent the visual appearance of images, *strel* function was applied to create a disk-shaped structuring element, at the radius of 30 pixels [25]. Then, *imtophat* function was applied to remove uneven background illumination from an image with a dark background [26]. Images were then processed by median filtering (*medfilt2* function) in two dimensions: each output pixel equals to the average of the median value in a 3 × 3 adjacent corresponding pixels in the input image. By applying the median filtering, the fluctuations of signal during acquisition process can be removed, without compromising the sharpness of the image [27]. To enhance the contrast of the grayscale images, *adapthisteq* function was then used to adjust images, followed by smoothing the images using low-pass Wiener filter. The Wiener filter removes the constant additive noise (Gaussian white noise) to preserve edges or other high-frequency parts of an image [27].

### Image analysis–image segmentation and quantification of morphological features

The overall goal of image segmentation was to identify boundaries of each *E*. *coli* cell and measure changes of cellular areas and shapes. Segmented images were then converted to binary images based on a global threshold. Binarization of images was a key step toward quantitative interpretation of image data from computer-aided machine vision, as opposed to human vision. To be more specific, *graythresh* function was applied to produce a global threshold. The *graythresh* function was based on Otsu’s method which minimizes the intraclass variance between black and white pixels [28]. Then, the binary images were created based on the threshold generated from *graythresh* function. Basically, any point (x, y) in the input image which has the function of f (x, y) ≥T is going to be designated as a white pixel (object) while other point which has f (x, y) < T is going to be served as a black pixel (background). Generation of binary image was created by *imbinarize* function [27]. To further ease the process of image analysis, *imfill* function was applied to fill holes in the input binary image. The holes are defined as background pixels (black) that are surrounded by the object pixels (white) [27]. In several cases, some small particles may show up as SYBR green I can occasionally stain non-specifically on anodisc. The function *bwareaopen* was then applied to remove any particles that are less than 300 pixels [29].

After binary images were created, modified and cleaned, quantification of morphological features was achieved using *regionprops* function. Bacteria did not undergo phage induced lysis has a rod shape, with confined DNA region. In comparison, bacteria that underwent phage lysis appeared as round shape, with diffuse distribution of released eDNA stained by SYBR Green I. To statistically and objectively compare the cellular morphology change and release of eDNA, “area” and “eccentricity” were chosen as two parameters representing the relative cellular morphology change. The property ‘area’ refers to the actual number of pixels in an object region which can be correlated with eDNA release (bigger area, more eDNA release) and the property “eccentricity” refers to the shape of an ellipse, and it measures the distance ratio between the foci of the ellipse and the length of its major axis. The ratio value of “eccentricity” is between 0 to 1 where 0 refers to a circle and 1 refers to a line segment. “Eccentricity” parameter is associated with the shape of bacteria where the rod shape bacteria have eccentricity closer to 1 while lysed round bacteria shape has eccentricity closer to 0.

The fluorescence intensity distribution was another morphological property chosen to detect the cellular shape change due to the release of the internal DNA which was represented by the full width at half maximum (FWHM) of the intensity distribution curve. For image analysis, all fluorescence images were analyzed by the ImageJ. The FWHM_Line plug-in was used to generate two-dimensional graph of the intensities of pixels along a 100-pixel line that was drawn across the cross-section of individual cells. The intensity distribution data was fitted to the Gaussian distribution and the full width at half maximum was measured.

### Statistical analysis

All conditions that were subjected to area, eccentricity, and intensity distribution analysis were conducted in triplicate and from each replicate, 5–8 images per replicate were selected for analysis. The total number of 15–20 images were analyzed, containing total objects equal or larger than 100 (N ≥100). Area, eccentricity, and FWHM value were generated from each image and averaged. Mean value of area, eccentricity, and FWHM were statistically compared between phage-lysed images and their corresponding negative controls, using SAS 9.4 (SAS Institute Inc., Cary, NC) by Tukey’s honest significant difference (HSD) test to compare the means.

## Results

### *E*. *coli* cellular morphological change and eDNA release during LTCL

**Fig 2:**
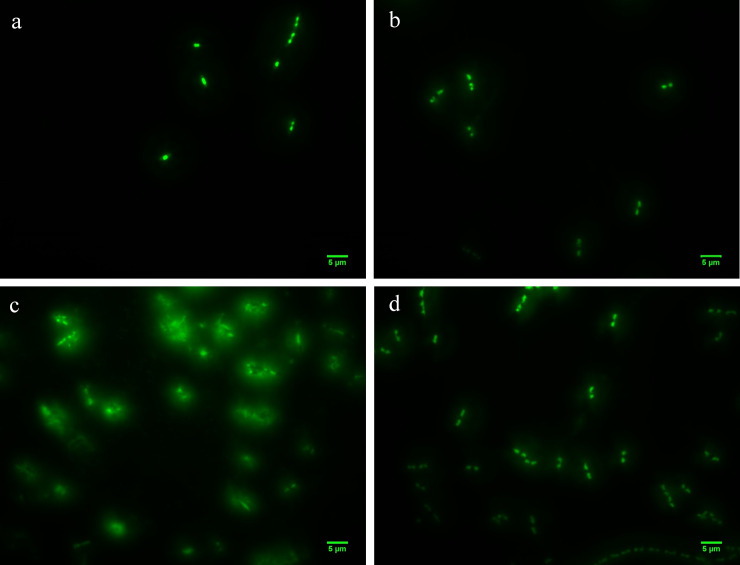
Bacteria morphology change during phage-induced lysis while co-incubating *E*. *coli* and T7 phages for 2 and 3 hours. a) cell morphology when co-incubating *E*. *coli* and T7 phages for 2 hours. b) cell morphology when incubating just *E*. *coli* for 2 hours. c) cell morphology when co-incubating *E*. *coli* and T7 phages for 3 hours. d) cell morphology when incubating just *E*. *coli* for 3 hours.

The overall goal of this set of experiments was to develop imaging-based framework for characterizing changes in cellular morphology and release of eDNA following the lysis of *E*. *coli* cells with phages during LTCL incubation conditions. As represented in Fig 2, the initial phage concentration was 10^2^ PFU/ml and *E*. *coli* BL21 was at 10^3^ CFU/ml in TSB. The phage-*E*. *coli* mixture was then incubated for 2 and 3 hours at 37°C and images were captured as shown in Fig 2A and 2C, respectively. In comparison, the negative control without adding phages were also performed under the same conditions, as shown in Fig 2B and 2D, respectively. As illustrated in Fig 2A and 2B, co-incubating phage and bacteria for 2 hours did result in observed cellular morphology change or eDNA release. After 3 hours, cell lysis was observed, as shown in Fig 2C, with the transition from rod-shaped cells to round-shaped cells. The release of eDNA, as seen in Fig 2C, was also noticeable; appearing as enlarged cellular areas of fluorescent signal with a fuzzy boundary which increased the intensity distribution of the cell particles. Neither cell morphological changes nor the appearance of any eDNA signal was evident for the negative controls (Fig 2D).

### Limit of detection of *E*. *coli* based on morphological change and eDNA release through LTCL

**Fig 3:**
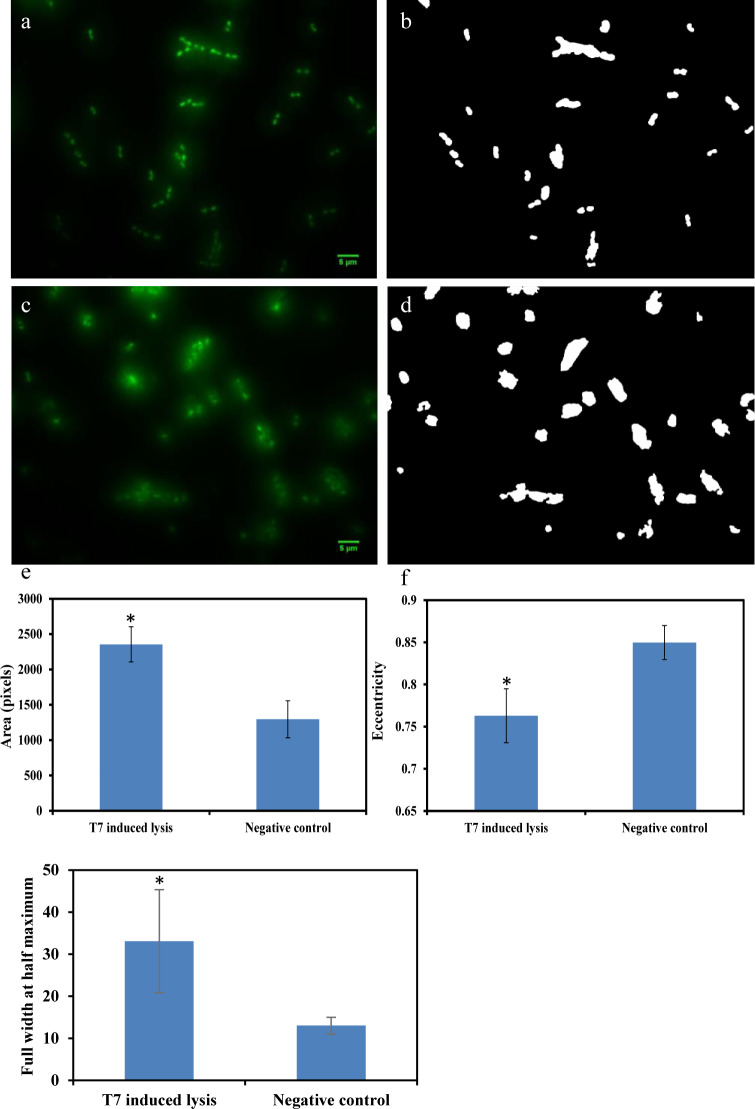
Detection of 10^3^ CFU/ml *E*. *coli* through LTCL with 10^2^ PFU/ml T7 phage. a) negative control which contains only *E*. *coli* growing for 3 hours. b) binary image of a). c) phage induced lysis while co-incubating with *E*. *coli*. d) binary image of c). e) comparison of area values extracted from b) and d). f) comparison of eccentricity values extracted from b) and d). g) comparison of the full width at half maximum extracted from a) and c). *indicated significant difference (P < 0.05).

In this approach, *E*. *coli* were co-incubated with low phage titer to allow initial propagation of bacteria, followed by phage lysis. Area, eccentricity and the FWHM parameter, taken together, were evaluated to distinguish “intact” *E*. *coli* cells from *E*. *coli* that are “lysed by phages”. As indicated in Fig 3, *E*. *coli* at 10^3^ CFU/ml can be detected based on changes in the selected morphological particles following incubation with 10^2^ PFU/ml of T7 phages. Fig 3A and 3B illustrate the microscopic image and binary image of negative control (only *E*. *coli* was incubated for 3 hours). In comparison, Fig 3C and 3D illustrate microscopic image and binary picture of *E*. *coli*-T7 phage for LTCL incubation condition. Based on image analysis methods as described in the previous section, area and eccentricity values for *E*. *coli* lysed by T7 phages demonstrate significant differences compared to the control (P < 0.05). In summary, *E*. *coli* lysed by T7 phages revealed significant eDNA release and changes from a rod to circular morphology.

Detection of *E*. *coli* at initial concentration of 10^2^ and 10^4^ CFU/ml were also conducted similarly using the LTCL incubation conditions. Resultant images were shown, analyzed and compared in S1 and S2 Figs, respectively. As shown in S1 Fig, no significant difference (P > 0.05) was observed for all parameters (area, eccentricity and FWHM) between images from samples of *E*. *coli* incubated with the T7 phage and negative controls, indicating *E*. *coli* at initial concentration of 10^2^ CFU/ml cannot be detected using LTCL incubation conditions and image analysis, due to lack of significant cell lysis. In comparison, *E*. *coli* at initial concentration of 10^4^ CFU/ml can be detected through LTCL, image analysis and comparison of area, eccentricity and FWHM parameters (S2 Fig).

### Characterization of *E*. *coli* morphological change and eDNA release through HTTL

**Fig 4:**
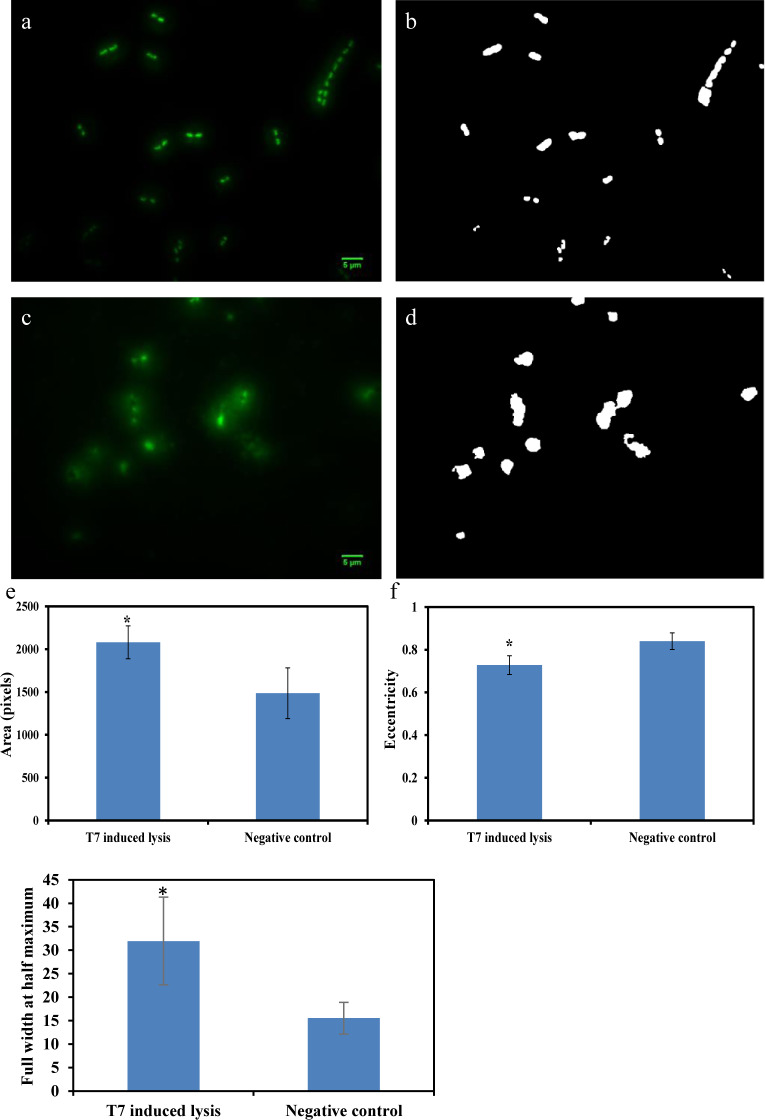
Detection of 10 CFU/ml *E*. *coli* through enrichment and HTTL. a) negative control which contains only *E*. *coli* growing for 5 hours. b) binary image of a). c) phage induced lysis after *E*. *coli* enrichment. d) binary image of c). e) comparison of area values extracted from b) and d). f) comparison of eccentricity values extracted from b) and d). g) comparison of the full width at half maximum extracted from a) and c). *indicated significant difference (P < 0.05).

*E*. *coli* at 10 CFU/ml can be detected by HTTL as shown in Fig 4. Fig 4A and 4B indicate microscopic image and binary picture of negative control where *E*. *coli* was incubated for 5 hours. In comparison, Fig 4C and 4D represents microscopic image and binary picture of *E*. *coli* lysed by T7 phage through HTTL. Quantitative image analysis comparisons between Fig 4B and 4D are presented in Fig 4E–4G. To be specific, area difference was compared and summarized in Fig 4E where *E*. *coli* cells lysed by T7 phage demonstrated larger stained area due to DNA leakage. Shape changes were also observed in Fig 4F where more circular morphology of *E*. *coli* were generated after T7 phage induced lysis. In addition, diffused fluorescent signal intensity from T7 phage lysed *E*. *coli* cells were observed in Fig 4G based on quantification of FWHM parameters compared to control. In summary, all area, eccentricity and FWHM parameters indicated statistical difference between HTTL and negative controls (P < 0.05), indicating *E*. *coli* at initial concentration of 10 CFU/ml can be detected through HTTL and image analysis.

Detection of *E*. *coli* at initial concentration 10^2^ and 10^3^ CFU/ml were also achieved through HTTL, image analysis and comparison of area, eccentricity and FWHM was presented in S3 and S4 Figs, respectively.

### Detection of *E*. *coli* at 10 CFU/ml in artificial wash water and coconut water

HTTL was selected as a preferred lysis approach for rapid detection of *E*. *coli* in realistic food samples, as HTTL demonstrates lower detection limit (10 CFU/ml) comparing to LTCL (10^3^ CFU/ml) as previously described based on area, eccentricity and FWHM parameters.

**Fig 5:**
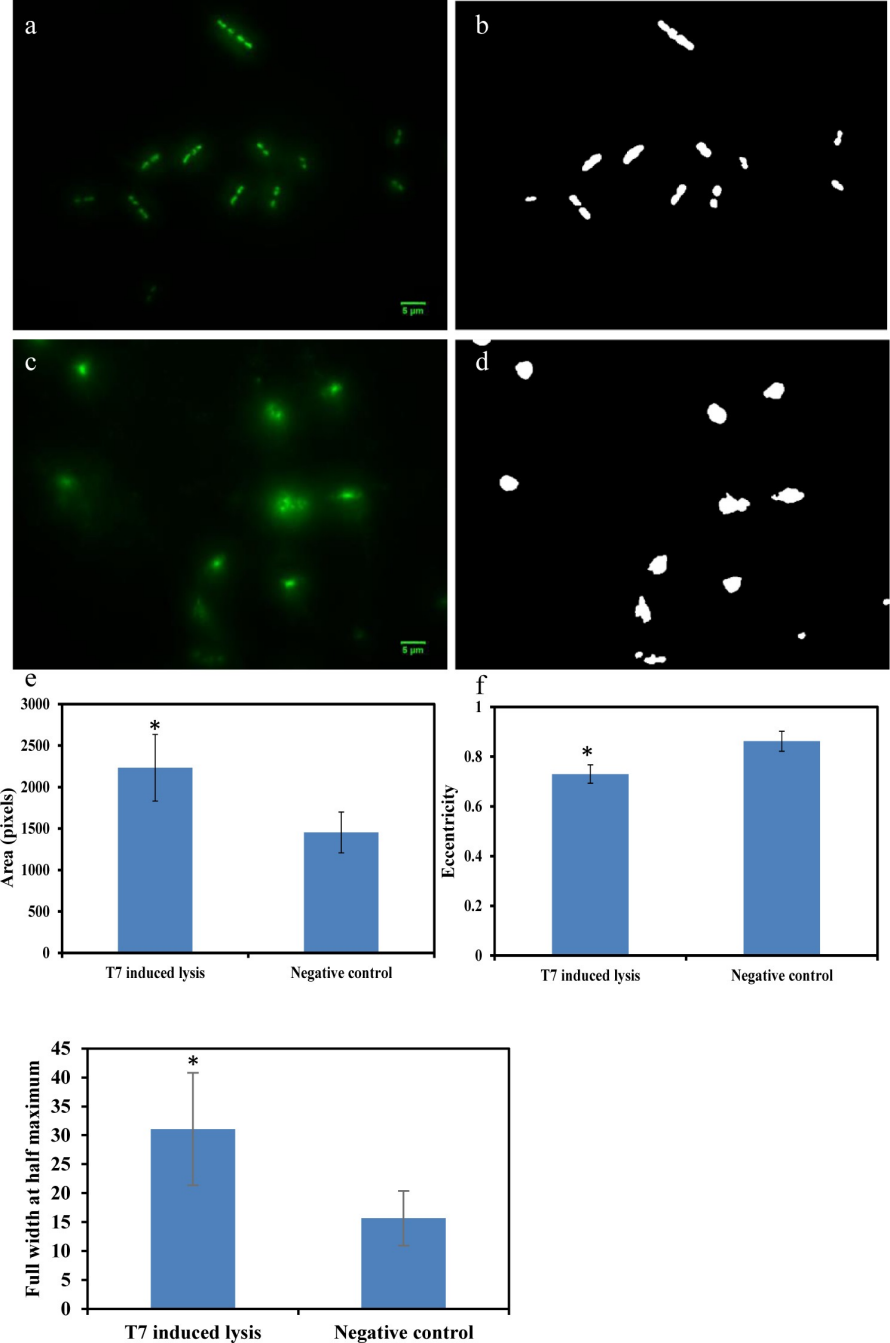
Detection of 10 CFU/ml *E*. *coli* through enrichment and HTTL in artificial wash water. a) negative control which contains only *E*. *coli* growing for 5 hours. b) binary image of a). c) phage induced lysis after *E*. *coli* enrichment. d) binary image of c). e) comparison of area values extracted from b) and d). f) comparison of eccentricity values extracted from b) and d). g) comparison of the full width at half maximum extracted from a) and c). *indicated significant difference (P < 0.05).

As shown in Fig 5, *E*. *coli* at 10 CFU/ml can be detected through HTTL coupled with image acquisition and analysis. Fig 5A and 5B indicate microscopic image and binary picture of negative control where *E*. *coli* was enriched in artificial wash water-TSB for 5 hours. In comparison, Fig 5C and 5D represents microscopic image and binary picture of *E*. *coli* lysed by T7 phage through HTTL after enrichment. Area, eccentricity and FWHM parameters were extracted and statistically compared in Fig 5E, 5F and 5G. As mentioned in the previous section, larger staining area, smaller eccentricity (more circular morphology) and larger FWHM (diffuse fluorescence signal) values indicated the lysis of *E*. *coli* cells upon incubation with T7 phages. In summary, based on significant differences in quantitative image analysis parameters, results indicated *E*. *coli* at 10 CFU/ml can be detected through HTTL and image analysis in simulated wash water samples.

**Fig 6:**
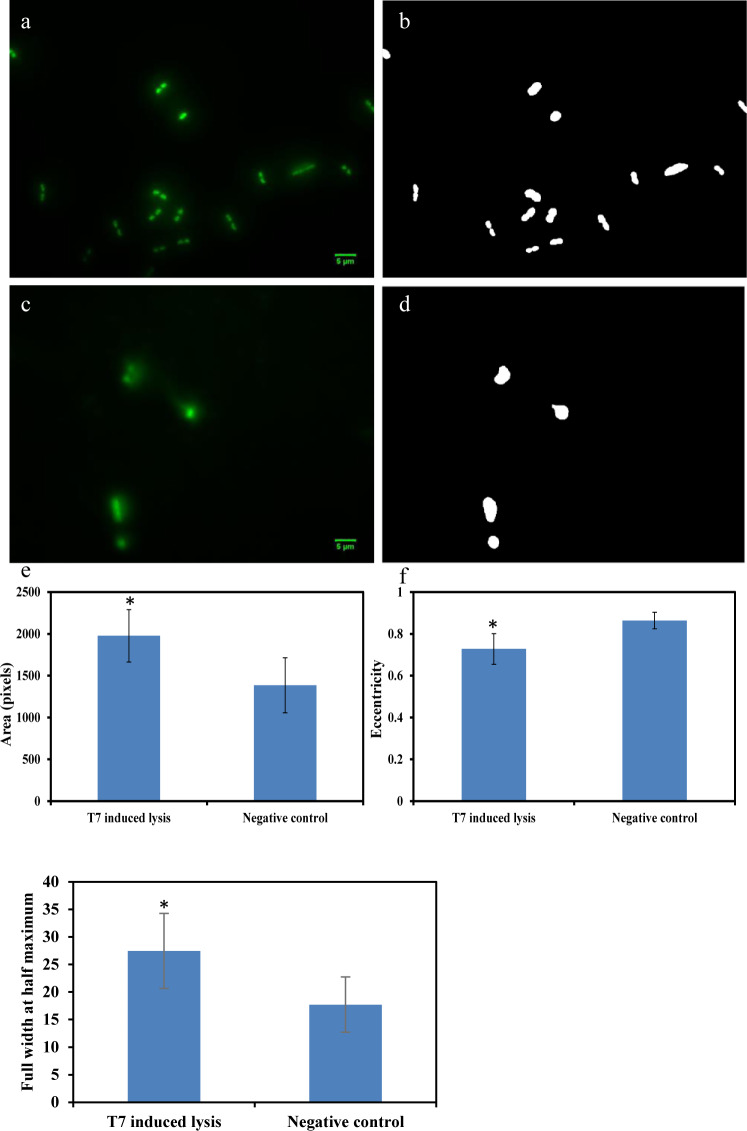
Detection of 10 CFU/ml *E*. *coli* through enrichment and HTTL in coconut water. a) negative control which contains only *E*. *coli* growing for 5 hours. b) binary image of a). c) phage induced lysis after *E*. *coli* enrichment. d) binary image of c). e) comparison of area values extracted from b) and d). f) comparison of eccentricity values extracted from b) and d). g) comparison of the full width at half maximum extracted from a) and c). *indicated significant difference (P < 0.05).

Similar experiments were conducted in coconut water and the resultant images, binary pictures and parameter analysis are shown in Fig 6. *E*. *coli* at 10 CFU/ml can be detected through HTTL and image analysis in coconut water. The proposed novel detection method is also validated in the spinach wash water, detailed data analysis and results are included in the supporting information.

## Discussion

The study illustrates a straightforward, rapid and cost-effective biosensing strategy to detect *E*. *coli* in simulated wash water, coconut water and spinach wash water. This concept can be potentially expanded to detect other bacteria of interest of food safety using host-specific lytic phages. The principle of the proposed biosensing strategy is rather straightforward: it focuses on morphological changes of host cells and the release of eDNA upon host cell lysis. As far as we are aware, this is the first study to describe combining morphological changes, eDNA release and image analysis to achieve rapid detection of *E*. *coli* in realistic food matrices. The application of imaging and image analysis as a detection approach has also been applied in BARDOT–a bacterial rapid detection using optical scattering technology. In BARDOT, a laser beam would usually pass through the bacterial colony to analyze 3D morphological and optical characteristics to generate optical “fingerprint”. Our proposed biosensing strategy using a similar optical imaging and image analysis approach enables significantly rapid detection compared with colony forming assays used for BARDOT. For instance, it took at least 6–8 hours or 24–36 hours to achieve detectable *Bacillus* and *Listeria* colonies, respectively [30–32]. In comparison, the rapidity of our proposed biosensing strategy is indicated in Table 1 and as shown in Table 1, the total amount of time for this proposed biosensing strategy is less than 8 hours. This is a time frame that fits within a typical industrial shift packaging perishable produce products. It could be argued that this approach has not eliminated the initial enrichment process as it adds significant time to the overall procedure but using the current imaging analysis approach was not possible without compromising the detection limit. Several other rapid detection methods based on PCR, phage and immunoassay still use enrichment as a pre-detection step to enhance the performance of the methods [14,33–37]. There is another acceptable reason to consider enrichment. It has been noted that bacterial cells that are naturally in the food/environment display an injured phenotype due to various stresses, which may reduce sensitivity of bacteria to phages during both infection and propagation step. The enrichment step could resuscitate injured cells thereby making them susceptible to phage infection and therefore, detectable by the proposed method.

**Table 1:** Total time and cost analysis for liquid food matrix (25 ml) using proposed biosensing strategy to detect *E*. *coli*. All prices were quoted from the suppliers listed in the material and method section.

Steps	Time	Supplies Used	Cost
Enrichment	5 hours	Food Sample, TSB broth	$0.20
Lysis	30 min	Phage	N/A
Filtration	30 min	Anodisc	$4.83
Staining	20 min	SYBR green	$0.03
Imaging	30 min	Microscopic slide, antifading reagent, cover slip	$0.44
Image Analysis	10 min	Matlab Software	N/A
Total	**7 hours**		**$5.50**

The biosensing strategy developed in this study is also cost-effective. Table 1 summarizes the cost of the detection method, which is around $5.50 per sample. The major expense of the biosensing strategy is anodisc ($4.40), which could potentially be replaced by another lower-cost material that yields similar performance with low fluorescence background upon staining with SYBR Green I.

Some of the potential challenges in translating the results of this study to field application may include: precise control on phage activity and presence of other bacteria with circular morphology in a food matrix. Based on our observations, phage activity needs to be controlled precisely to achieve timely detection as older batch of phage due to reduced phage titer or weaker infectious potency, may take longer time for lysis. To partially resolve the phage activity inconsistence, new batch of phages were prepared every two weeks. Another potential limitation of the biosensing strategy lies in the background microflora. Presence of coccoid bacteria, such as a *Staphylococcus* spp., may influence the eccentricity value during image analysis. The presence of bacteria that are significantly larger than *E*. *coli*, such as some of *Pseudomonas* spp., could also influence the calculation of area. The potential challenge from background microflora was partially addressed in the manuscript by simultaneously investigating changes in both area and eccentricity parameters, and validation in realistic spinach wash water samples to conclude the presence/absence of *E*. *coli*. ‘Intensity distribution’ which is the fluorescence intensity as a function of distance of the line cross-sectioned through the centroid of a bacterium cell is another parameter that is able to detect bacterial cell lysis. The lysis of cell membrane results in diffused distribution of the DNA. This diffuse distribution increases the width at half maximum while un-lysed cell showed focused DNA staining and reduced full width at half maximum.

The manuscript also describes two modes of lysis, LTCL and HTTL. Both lysis pathways have advantages and disadvantages. LTCL provides easier sample preparation as phages and *E*. *coli* were co-incubated together, without additional step before filtration. This mode of sample preparation for the detection of target bacteria using phages has also been documented in several studies [7,21]. However, major drawbacks for LTCL were noticed as the relative insensitive detection limit at10^3^ CFU/ml. In comparison, HTTL provides better detection limit in our current study at 10 CFU/ml, even though sample preparation requires both enrichment and phage addition at two different steps. To conclude, HTTL lysis mode is a better approach in our biosensing strategy.

## Conclusion

In conclusion, this study demonstrates a straightforward, rapid and cost-effective biosensing strategy to be developed which focuses on host cell morphology change and eDNA release, followed by automated imaging acquisition and analysis. The proposed method has been tested to rapidly detect *E*. *coli* at 10 CFU/ml within 8 hours of entire process. The method has also been validated in three different realistic matrices—artificial fresh produce wash water, coconut water and spinach wash water. Future research may be conducted to include a variety of background microflora and to test the robustness of the proposed biosensing strategy in a greater variety of food matrices.

## Supporting information

S1 Fig Detection of 10^2^ CFU/ml *E*. *coli* through LTCL with 10^2^ PFU/ml T7 phage for 4 hours. a) negative control which contains only *E*. *coli* growing for 4 hours. b) binary image of a). c) phage induced lysis while co-incubating with *E*. *coli*. d) binary image of c). e) comparison of area values extracted from b) and d). f) comparison of eccentricity values extracted from b) and d). g) comparison of the full width at half maximum extracted from a) and c).
(DOCX)

S2 Fig Detection of 10^4^ CFU/ml *E*. *coli* through LTCL with 10^2^ PFU/ml T7 phage for 2 hours. a) negative control which contains only *E*. *coli* growing for 2 hours. b) binary image of a). c) phage induced lysis while co-incubating with *E*. *coli*. d) binary image of c). e) comparison of area values extracted from b) and d). f) comparison of eccentricity values extracted from b) and d). g) comparison of the full width at half maximum extracted from a) and c). *indicated significant difference (P < 0.05).
(DOCX)

S3 Fig Detection of 10^2^ CFU/ml *E*. *coli* through 4 hours enrichment and HTTL. a) negative control which contains only *E*. *coli* growing for 4 hours. b) binary image of a). c) phage induced lysis after *E*. *coli* enrichment. d) binary image of c). e) comparison of area values extracted from b) and d). f) comparison of eccentricity values extracted from b) and d). g) comparison of the full width at half maximum extracted from a) and c). *indicated significant difference (P < 0.05).
(DOCX)

S4 Fig Detection of 10^3^ CFU/ml *E*. *coli* through 2 hours enrichment and HTTL. a) negative control which contains only *E*. *coli* growing for 2 hours a) negative control which contains only *E*. *coli* growing for 2 hours. b) binary image of a). c) phage induced lysis after *E*. *coli* enrichment. d) binary image of c). e) comparison of area values extracted from b) and d). f) comparison of eccentricity values extracted from b) and d). g) comparison of the full width at half maximum extracted from a) and c). *indicated significant difference (P < 0.05).
(DOCX)

S5 Fig Detection of 10^3^ CFU/ml *E*. *coli* through 3 hours enrichment and HTTL in spinach wash water. a) negative control image which contains only *E*. *coli* growing for 3 hours. b) phage induced lysis after *E*. *coli* enrichment. c) comparison of area, d) comparison of eccentricity and e) comparison of full width at half maximum values between *E*. *coli* cells with or without T7 induced lysis. *indicated significant difference (P < 0.05).
(DOCX)

S6 Fig Fluorescence images of various non-*E*. *coli* bacterial cells with and without T7 phage infection. a) *Bacillus subtilis* without infection, b) *Bacillus subtilis* with infection, c) *Lactobacillus casei* without infection, d) *Lactobacillus casei* with infection, e) *Listeria innocua* without infection, f) *Listeria innocua* with infection, g) *Pseudomonas fluorescens* without infection and h) *Pseudomonas fluorescens* with infection.
(DOCX)

S1 Data (DOCX)
