# Supplementary material for: Rapid detection of Escherichia coli using bacteriophage-induced lysis and image analysis
Source: PLoS One. 2020 Jun 5;15(6):e0233853. doi: 10.1371/journal.pone.0233853 (PMC7274428; doi:10.1371/journal.pone.0233853)
Supplement: S2 Fig — a) negative control which contains only E. coli growing for 2 hours. b) binary image of a). c) phage induced lysis while co-incubating with E. coli. d) binary image of c). e) comparison of area values extracted from b) and d). f) comparison of eccentricity values extracted from b) and d). g) comparison of the full width at half maximum extracted from a) and c). *indicated significant difference (P < 0.05). (DOCX) [file pone.0233853.s002.docx]

**S2 Fig.** Detection of 10^4^ CFU/ml *E. coli* through LTCL with 10^2^ PFU/ml T7 phage for 2 hours. a) negative control which contains only *E. coli* growing for 2 hours. b) binary image of a). c) phage induced lysis while co-incubating with *E. coli*. d) binary image of c). e) comparison of area values extracted from b) and d). f) comparison of eccentricity values extracted from b) and d). g) comparison of the full width at half maximum extracted from a) and c). *indicated significant difference (P < 0.05).
