# Supplementary material for: Rapid detection of Escherichia coli using bacteriophage-induced lysis and image analysis
Source: PLoS One. 2020 Jun 5;15(6):e0233853. doi: 10.1371/journal.pone.0233853 (PMC7274428; doi:10.1371/journal.pone.0233853)
Supplement: S5 Fig — a) negative control image which contains only E. coli growing for 3 hours. b) phage induced lysis after E. coli enrichment. c) comparison of area, d) comparison of eccentricity and e) comparison of full width at half maximum values between E. coli cells with or without T7 induced lysis. *indicated significant difference (P < 0.05). (DOCX) [file pone.0233853.s005.docx]

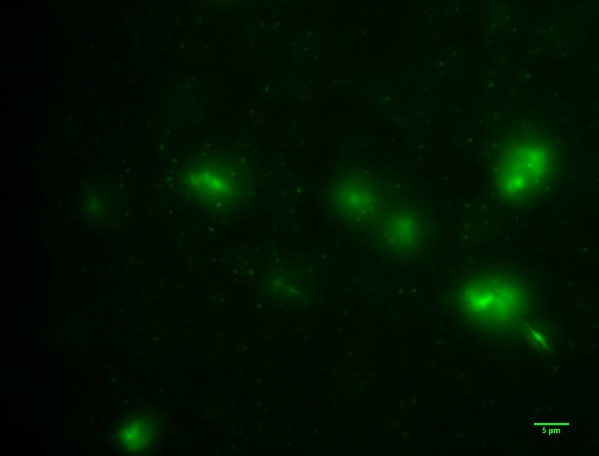

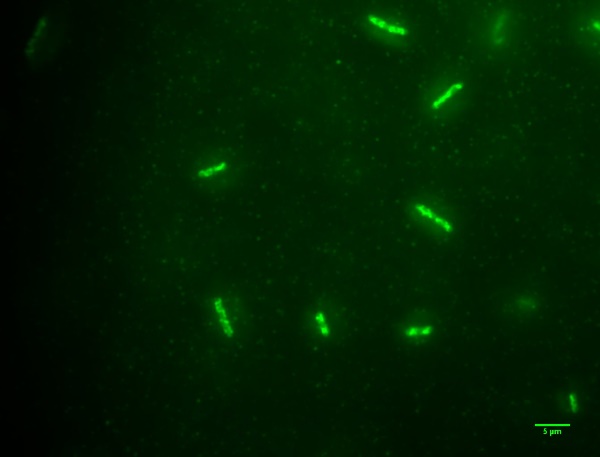


b

a

d

c


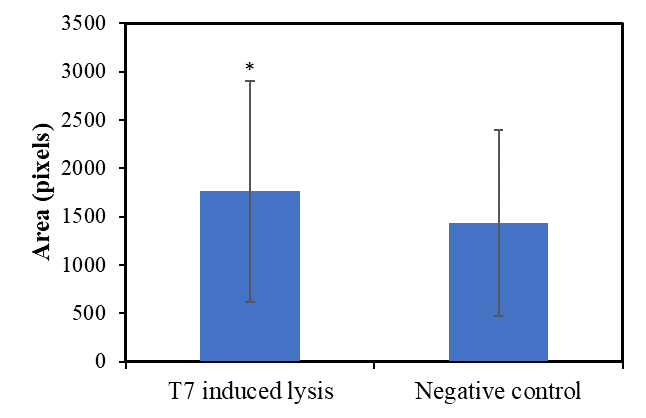

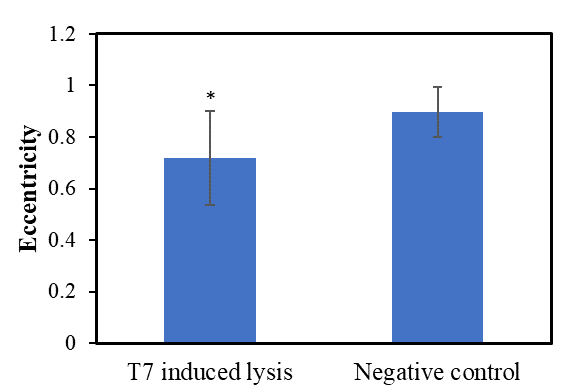


e


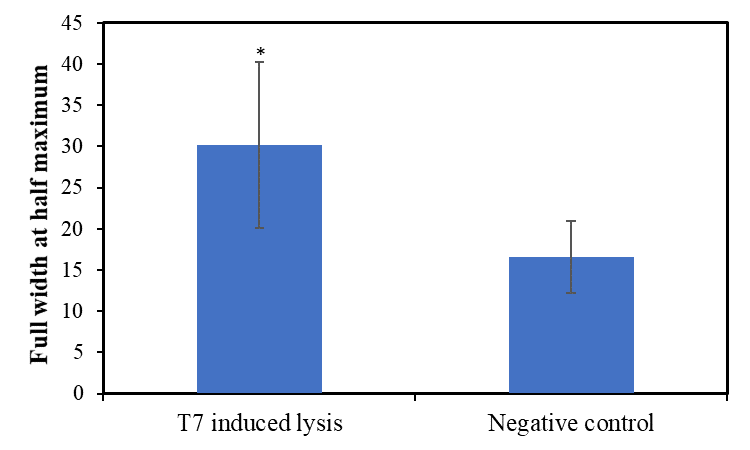


**S5 Fig.** Detection of 10^3^ CFU/ml *E. coli* through 3 hours enrichment and HTTL in spinach wash water. a) negative control image which contains only *E. coli* growing for 3 hours. b) phage induced lysis after *E. coli* enrichment. c) comparison of area, d) comparison of eccentricity and e) comparison of full width at half maximum values between *E. coli* cells with or without T7 induced lysis. *indicated significant difference (P < 0.05).
