# Supplementary material for: Rapid detection of Escherichia coli using bacteriophage-induced lysis and image analysis
Source: PLoS One. 2020 Jun 5;15(6):e0233853. doi: 10.1371/journal.pone.0233853 (PMC7274428; doi:10.1371/journal.pone.0233853)
Supplement: S6 Fig — a) Bacillus subtilis without infection, b) Bacillus subtilis with infection, c) Lactobacillus casei without infection, d) Lactobacillus casei with infection, e) Listeria innocua without infection, f) Listeria innocua with infection, g) Pseudomonas fluorescens without infection and h) Pseudomonas fluorescens with infection. (DOCX) [file pone.0233853.s006.docx]

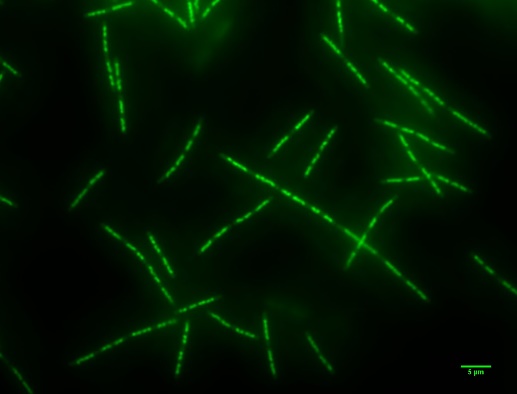

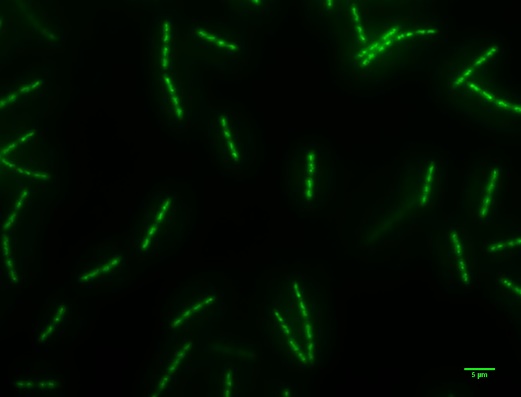


c


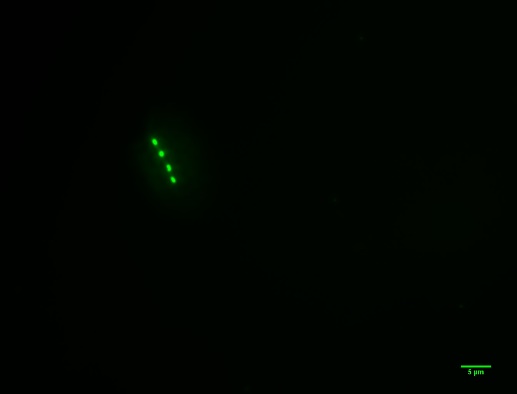


d

a

b


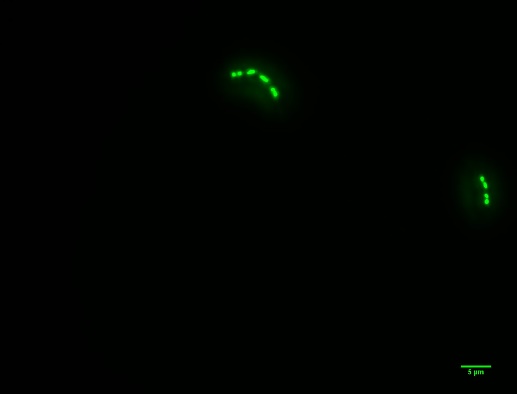


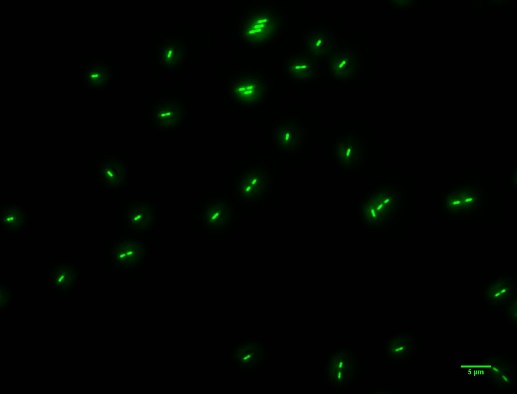


e


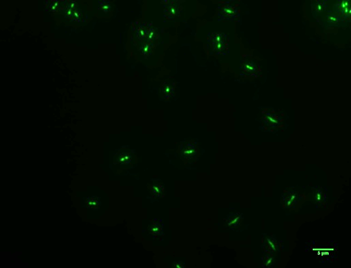


f


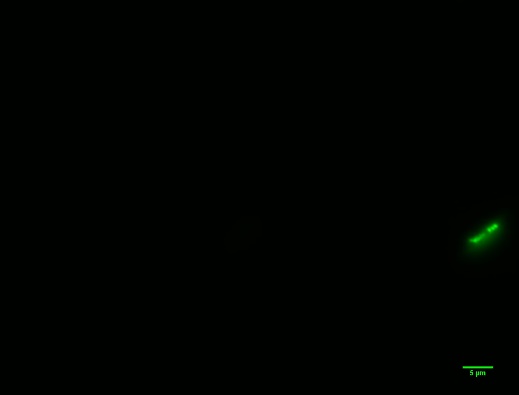


h

g


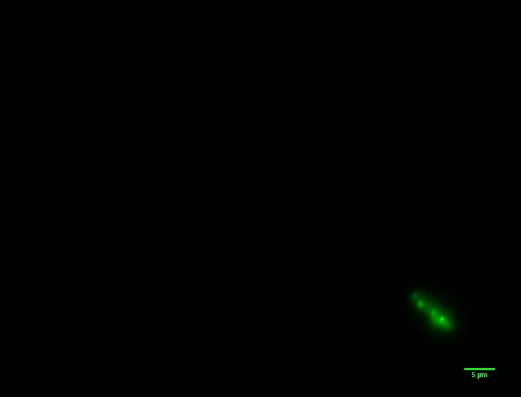


**S6 Fig.** Fluorescence images of various non-*E. coli* bacterial cells with and without T7 phage infection. a) *Bacillus subtilis* without infection, b) *Bacillus subtilis* with infection, c) *Lactobacillus casei* without infection, d) *Lactobacillus casei* with infection, e) *Listeria innocua* without infection, f) *Listeria innocua* with infection, g) *Pseudomonas fluorescens* without infection and h) *Pseudomonas fluorescens* with infection.
