# Supplementary material for: Rapid detection of Escherichia coli using bacteriophage-induced lysis and image analysis
Source: PLoS One. 2020 Jun 5;15(6):e0233853. doi: 10.1371/journal.pone.0233853 (PMC7274428; doi:10.1371/journal.pone.0233853)
Supplement: S1 Data — (DOCX) [file pone.0233853.s007.docx]

**Materials and Methods**

**Validation of biosensing approach in spinach wash water.** Spinach wash water was selected to represent complex unsterilized food matrices that contain background microbiota and plants debris. Briefly, spinach water was prepared by blending baby spinach leaves with autoclaved milli Q water at 1:10 wt/vol using sterile blender. The mixture was blended for 30 seconds twice at maximum speed. The blended solution was then transferred to 50 ml centrifuge tubes and centrifuged at 11,000 × *g* for 10 minutes. Supernatant was collected before another centrifugation at the same condition. The final supernatant was collected [1]. The chemical oxygen demand (COD) value of the spinach water was proximately 3000 mg/ml. The spinach wash water was then mixed with double concentrated tryptic soy broth (TSB) which has a COD value of proximately 28,000 mg/ml at the ratio of 1:1. Detection of target bacteria using this method with HTTL (high titer two-step lysis) condition was performed in the spinach water-TSB solution. *E. coli* BL21 (10^3^ CFU/ml) was inoculated followed by enrichment at 37°C for 3 hours. T7 phage with the final concentration of 10^7^ PFU/ml was added and incubated at the same condition for 30 minutes. Then, the mixture was filtered, stained, and visualized using fluorescence microscope as described in the materials and methods. The total number of 25 images were analyzed, containing total objects larger than 150 (N > 150) for each condition.

**Specificity test of T7 phages against other bacterial indicators.** The specificity of this detection method with HTTL condition was tested with several food and agriculture related bacterial strains including Gram-positive *Bacillus subtilis,* *Lactobacillus casei*, *Listeria innocua*, and Gram-negative *Pseudomonas fluorescens*. *B. subtilis* are commonly found at the upper layers of the soil and plant rhizosphere [2]. *L. casei* represent lactic acid bacteria that are widely distributed in nature and are easily isolated from mucous surfaces of mammals, green plants, milk, and fermenting foods [3]. *L. innocua* is a nonpathogenic bacterial strain which usually serves as a surrogate of *Listeria monocytogenes* [4]. *P. fluorescens* is a common bacterial strain causing food spoilage [5].

Bacteria with an initial concentration of 10^3^ CFU/ml were inoculated in 10 ml of TSB and incubated at 37°C for 5 hours to obtain proper bacterial cells concentration. Then, bacterial cultures were infected with T7 phage (10^7^ PFU/ml) for 30 minutes. After infection, the samples were proceeded to fluorescence imaging as described in the materials and methods.

**Results**

**Detection of *E. coli* in spinach wash water.** A representative fluorescence images of bacterial cells initially inoculated in spinach wash water with and without T7 phage infection are shown in S5 Fig. Bacterial cells inoculated in spinach wash water mixed with TSB and enriched for 3 hours are infected with T7 phage. The results in S5 Fig illustrate the images of control bacteria without phage infection (S5 Fig a) and with phage infections (S5 Fig b). The total number of 25 images were analyzed, containing total objects larger than 150 (N > 150) for each condition. The results demonstrate that upon infection, significant morphological changes are observed in the target bacteria. The changes include enlargement of the size, diffused edge, and more circular morphology compared to the controls. These changes in structural parameters indicate the lysis of cells caused by phage T7 infection. In order to quantitatively measure the change in the cellular structure, the fluorescence images were analyzed using image processing. As shown in S5 Fig c and e, both area and FWHM of the lysed bacterial cells were significantly higher than those of the un-lysed cells. While the eccentricity of the lysed cells is significantly lower than those of un-lysed cells, indicating lysed cells have more circular morphology. These changes are similar to the changes observed in the case of artificial wash water and coconut water samples.

**Specificity test of T7 phages against other bacterial indicators.** S6 Fig shows fluorescence images of non-*E. coli* bacterial cell morphology, with or without T7 phage infection. According to the results, there was no difference in bacterial cell shape before and after T7 phage infection which indicates that there was no lysis of these non-*E. coli* cells by T7 phages. Therefore, we can conclude T7 phages have high specificity, and will not affect *Bacillus*, *Lactobacillus*, *Listeria* and *Pseudomonas* tested in our study.

g

g

g

g

**References**

1. Tilton L, Das G, Yang X, Wisuthiphaet N, Kennedy IM, Nitin N. Nanophotonic Device in Combination with Bacteriophages for Enhancing Detection Sensitivity of *Escherichia coli* in Simulated Wash Water. Anal Lett. 2019. doi:10.1080/00032719.2019.1604726

2. van Dijl JM, Hecker M. *Bacillus subtilis*: From soil bacterium to super-secreting cell factory. Microb Cell Fact. 2013;12: 1–6. doi:10.1186/1475-2859-12-3

3. Sannine WE, Muralidhara KS, Elliker PR, England DC. Lactic Acid Bacteria in Food and Health: a Review With Special Reference To Enteropathogenic *Escherichia coli* As Well As Certain Enteric Diseases and Their Treatment With Antibiotics and Lactobacilli1. J Milk Food Technol. 1972;35: 691–702. doi:10.4315/0022-2747-35.12.691

4. Friedly EC, Crandall PG, Ricke S, O’Bryan CA, Martin EM, Boyd LM. Identification of *Listeria innocua* surrogates for *Listeria monocytogenes* in hamburger patties. J Food Sci. 2008;73: 174–178. doi:10.1111/j.1750-3841.2008.00719.x

5. Martin NH, Murphy SC, Ralyea RD, Wiedmann M, Boor KJ. When cheese gets the blues: *Pseudomonas fluorescens* as the causative agent of cheese spoilage. J Dairy Sci. 2011;94: 3176–3183. doi:10.3168/jds.2011-4312
